# Lamivudine and Entecavir for Acute Hepatitis B: A Systematic Review and Meta-Analysis

**DOI:** 10.3390/v15112241

**Published:** 2023-11-10

**Authors:** Cesar Henriquez-Camacho, Ana Isabel Hijas-Gomez, Carlos Risco Risco, Maria Angeles Ruiz Lapuente, Rosa Escudero-Sanchez, Victor Moreno Cuerda

**Affiliations:** 1 Faculty of Medicine, Universidad Francisco de Vitoria (UFV), Hospital Universitario de Móstoles, 28935 Madrid, Spain; v.moreno.prof@ufv.es; 2 Agencia de Evaluación de Tecnologías Sanitarias (AETS), Instituto de Salud Carlos III, 28220 Madrid, Spain; 3 HM Hospitales, Hospital Universitario Sanchinarro, 28050 Madrid, Spain; 4 Centro de Vacunación Internacional, Sanidad Exterior Barcelona, 08002 Barcelona, Spain; 5 Hospital Universitario Ramón y Cajal, CIBERINFEC, ISIII-CIBER de Enfermedades Infecciosas, Instituto de Salud Carlos III, IRYCIS, Instituto Ramón y Cajal de Investigación Sanitaria, 28034 Madrid, Spain

**Keywords:** nucleoside analogue, lamivudine, entecavir, acute Hepatitis B, viral hepatitis

## Abstract

Background. Acute hepatitis B infection is associated with severe liver disease and chronic sequelae in some cases. The purpose of this review was to determine the efficacy of nucleoside analogues (NA) (lamivudine versus entecavir) compared to placebo or no intervention for treating acute primary HBV infection. Methods. A meta-analysis for drug intervention was performed, following a fixed-effect model. Randomized controlled trials (RCTs) and quasi-randomized studies that evaluated the outcomes of NA in acute hepatitis B infection were included. The following outcomes were considered: virological cure (PCR negative), elimination of acute infection (seroconversion of HBsAg), mortality, and serious adverse events. Results. Five trials with 627 adult participants with severe acute hepatitis B defined by biochemical and serologic parameters were included. Virological cure did not favor any intervention: OR 0.96, 95% CI 0.54 to 1.7 (*p* = 0.90), I2 = 58%. Seroconversion of HBsAg to negative favored placebo/standard-of-care compared to lamivudine: OR 0.54, 95% CI 0.33 to 0.9 (*p* = 0.02), I2 = 31%. The only trial that compared entecavir and lamivudine favored entecavir over lamivudine (OR: 3.64, 95% CI 1.31–10.13; 90 participants). Adverse events were mild. Conclusion. There is insufficient evidence that NA obtain superior efficacy compared with placebo/standard-of-care in patients with acute viral hepatitis, based on low quality evidence.

## 1. Introduction

Hepatitis B virus (HBV) infection, which is associated with chronic liver disease, affects almost 240 million people in the world [1,2]. The highest prevalence of chronic HBV is reported in sub-Saharan Africa and East Asia, where 5–10% of people are infected [3]. Less than 5% of immunocompetent adults who are infected will develop chronic infection, and 20–30% of them will have cirrhosis and/or liver cancer [3,4].

Risk factors for acquiring HBV include exposure to infected blood, perinatal transmission (mostly in the endemic regions), percutaneous or mucosal exposure to infected blood and various body fluids, sexual transmission, intravenous drugs, and open wounds [5,6]. In addition, infection may occur during medical, surgical, and dental procedures, tattooing, or through the use of contaminated razors and similar objects [3].

The incubation period of HBV is around 75 days (30 to 180 days), and the virus may be detected within 30 to 60 days after the infection [3]. Clinical manifestations of acute HBV infection range from asymptomatic to symptomatic. Among the symptomatic patients, approximately one-third of adults will develop prodromal constitutional symptoms including fever, fatigue, anorexia, general malaise, nausea, and body aches [7]. This phase is followed by the onset of jaundice and choluria. Fulminant liver failure is seen in 1% of patients [7].

More than 95% of immunocompetent adults with acute hepatitis recover spontaneously and treatment is not required [8]. Nowadays, the treatment for acute HBV infection is based on supportive management. Antiviral treatment is recommended for acute liver failure given its safety and the need of viral suppression if liver transplantation is required [8]. Also, some experts recommend treatment for patients with severe acute hepatitis B who have the following parameters: total bilirubin > 3 mg/dL or direct bilirubin > 1.5 mg/dL, international normalized ratio > 1.5, encephalopathy, or ascites [8]. The current National Institute of Health Consensus recommends treatment for patients who have acute liver failure, cirrhosis and clinical complications, cirrhosis or advanced fibrosis, and HBV after chemotherapy or immunosuppression [9].

Regarding therapy, nucleoside analogues (NA) are preferred in hepatitis B infection. IFN-α is contraindicated because of the risks of exacerbation of hepatitis and the frequent adverse effects [8]. Lamivudine, commonly called 3TC, is a nucleoside (deoxycytidine) analogue that inhibits reverse transcriptase of human immunodeficiency virus (HIV) and HBV [10,11]. Entecavir is another nucleoside (deoxyguanosine) analogue which inhibits HBV DNA polymerase, blocking both priming and elongation of the viral DNA replication [12].

NA have been used for treating chronic HBV infections [13], but the role of antiviral therapy in acute infection remains unsettled since the evidence available in some reports and trials is limited [14]. Although some trials have reported benefits with treatments that include nucleoside analogues (lamivudine or entecavir), there is not enough evidence to support antiviral treatment for acute HBV infection.

There is one systematic review published in 2017 [15], reporting that low or very low-quality evidence suggests that more patients in the lamivudine group progressed to chronic HBV infection compared to placebo/no intervention or entecavir. Some guidelines still recommend antivirals for acute severe HBV infection, and given the time elapsed, it is necessary to know if new evidence has been published in this regard.

The purpose of this review was to determine the efficacy of nucleoside analogues (lamivudine versus entecavir) compared to placebo or no intervention for treating acute primary HBV infection.

## 2. Materials and Methods

### 2.1. Selection Criteria

This systematic review and meta-analysis were registered in the International prospective register of systematic reviews (PROSPERO) with the reference CRD 42023432482, and it was conducted following the PRISMA 2020 statement criteria [16]. Randomized and quasi-randomized (predictable allocation) controlled trials that evaluated the outcomes of lamivudine or entecavir in acute hepatitis B infection were included. For inclusion, the studies were required to include immunocompetent adults with acute primary hepatitis B infection confirmed by the following serological tests: HBsAg and immunoglobulin M (IgM) antibody to the core antigen, HBcAg, and hepatitis B e antigen (HBeAg). We excluded studies which included immunosuppressed patients, coinfection with any other viral hepatitis (C, D, E), and chronic viral hepatitis or acute exacerbations of chronic HBV infection. The use of NA therapy (lamivudine or entecavir) was the only pharmacological intervention evaluated. The following outcomes were considered: elimination of infection (negative HBsAg, or IgM HBcAg, or HBeAg), mortality, and serious adverse events such as inpatient hospitalization or prolongation of existing hospitalization, persistent or significant disability/incapacity, or life-threatening states.

### 2.2. Search Strategy and Study Selection

We identified all relevant trials, regardless of their publication status (published, unpublished, in press, and in progress), using the search terms detailed in Supplementary Materials Table S1 in the following databases: MEDLINE (from January 1966 to May 2023) and Embase (from January 1980 to May 2023). The reference lists of included studies were identified by the mentioned methods. Two reviewers (AH, CR) checked the titles and abstracts of the literature independently, searching for potentially relevant articles. We retrieved the full reports of potentially relevant trials and applied the inclusion criteria using an eligibility form. The third author (CHC) resolved any disagreements. The eligible trials were scrutinized to ensure that each trial was included only once, eliminating duplicates. In the case of several publications which reported the same trial, the most recent was chosen. There were no restrictions based on language, sample size, age, gender, ethnicity, or duration of follow-up.

### 2.3. Data Extraction and Assessment of Risk of Bias

Two reviewers (RE and CH) independently extracted data regarding the inclusion criteria, outcome data, and adverse events. Any disagreement was resolved after evaluation by a third reviewer (CR). We tried to contact the trial authors to clarify missing data, but this was not successful. The risk of bias was assessed independently using a risk of bias Cochrane Collaboration form [17], while the third reviewer resolved any disagreements. Generation of allocation sequence and allocation concealment was categorized as adequate, unclear, or inadequate accordingly, using the criteria outlined in the Cochrane Handbook [18]. We considered the following domains: random sequence generation (selection bias), allocation concealment (selection bias), blinding of participants and personnel (performance bias), blinding of outcome assessment (detection bias), incomplete outcome data (attrition bias), selective outcome reporting (reporting bias), and other sources of bias. We classified each domain as being at ‘low’, ‘high’ or ‘unclear’ risk of bias. A ‘Risk of bias’ graph and a ‘Risk of bias’ summary were included in the analysis. Individual trial results were included in a funnel plot to assess the possibility that the pooled estimate may have been influenced by publication bias.

### 2.4. Outcome Assessment

The data extracted were dichotomous variables. We recorded the absolute number of events and participants in each group for all outcomes. Pooled OR with 95% CI was reported. We calculated the percentage of loss to follow-up in each group. We reported data about methodological quality of trials, characteristics of participants (age, gender, acute hepatitis B confirmed), characteristics of interventions, characteristics of outcome measures, date of trial, trial authors, location of trial, sponsor of trial (specified, known or unknown), design (described as randomized or not), interventions (treatment, days, doses), and outcomes (elimination of infection (negative HBsAg, or IgM HBcAg, or HBeAg), mortality, and serious adverse events). We calculated the percentage of loss to follow-up in each group.

### 2.5. Statistical Analysis

Review Manager (RevMan web Cochrane Collaboration) for data analysis was used. The odds ratio was calculated to pool dichotomous data across trials, and each result was presented with a 95% confidence interval. A meta-analysis for drug intervention was performed, following a fixed-effect model.

For the analysis of adverse events, the number of participants who experienced the adverse events were included. The odds ratio was used to pool adverse event data when the trials were sufficiently similar according to their definition. Data from trials that only reported the number of adverse events were not included, as it was possible that the same individual reported more than one adverse event.

Subgroup analysis of trials with high risk of bias compared to trials with low risk of bias was assessed using Chi2 test for subgroup differences.

Heterogeneity of treatment effects across trials was assessed inspecting the forest plots visually and calculating the Chi2 statistic for heterogeneity with a significance level at *p* < 0.1. We calculated the I2 statistic to quantify heterogeneity. If we detected high levels of heterogeneity (I^2^ > 75%), we planned to explore the possible sources in subgroup analyses.

A summary of findings table about quality of evidence was reported using GRADE method [19].

## 3. Results

**Figure 1:**
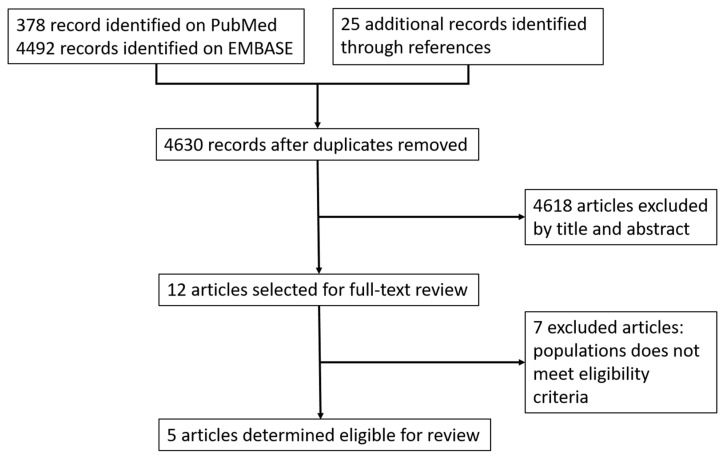
Study flow diagram.

The initial electronic search identified 378 references (PubMed), 4492 references (Embase), and 25 additional reports. After removing duplicates, 4618 references were excluded based on the title and abstract screening, and 12 were included after full-text screening. Finally, five trials [20,21,22,23,24] met the inclusion criteria for the meta-analysis (Figure 1). We excluded seven trials from the review because the population did not meet eligibility criteria and were not clinical trials [25,26,27,28,29,30,31].

### 3.1. Study Characteristics

Five trials (four randomized controlled trials and one quasi-randomized trial), including 627 adult participants, were assessed in this review. Two of them were conducted in Romania [20,24], one in Germany [23], one in China [22], and one in India [21]. None of the trials included patients with fulminant acute liver failure. None of the trials included immunocompromised patients. The median age of the participants ranged from 35 to 45 years, with a predominance of males. The inclusion criterion for patients of these trials was severe acute hepatitis B defined by biochemical and serologic parameters. One trial [20] did not define criteria for the severity of acute hepatitis B infection. See Table 1 and Table 2.

### 3.2. Interventions

In five trials lamivudine was compared with placebo or standard-of-care, and only one trial [24] compared three arms: entecavir, lamivudine, and placebo. The usual dose of lamivudine was 100 mg daily for 3–6 months, and the dose of entecavir was 0.5 mg daily for a maximum of six months. The analysis was per-protocol. The follow-up period ranged from 3 to 12 months.

### 3.3. Outcome Measures

The assessment of the outcome measures included seroconversion and undetected DNA [20,21,22], decrease in the bilirubin levels, seroconversion and undetected DNA [23], seroconversion, and decrease in the HBV viral load [24]. The trials assessed and reported different outcome measures, depending on the technique used. See Table 1.

### 3.4. Certainty of Evidence

The certainty of the evidence was low for all outcomes due to the high risk of bias in some studies, the imprecision of the findings due to the small sample size of the studies, and the high heterogeneity. See Supplementary Materials Tables S2–S4.

**Figure 2:**
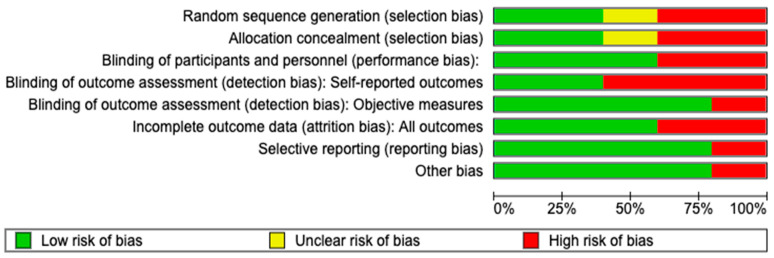
Risk of bias graph based on authors’ judgements of all included studies.

**Figure 3:**
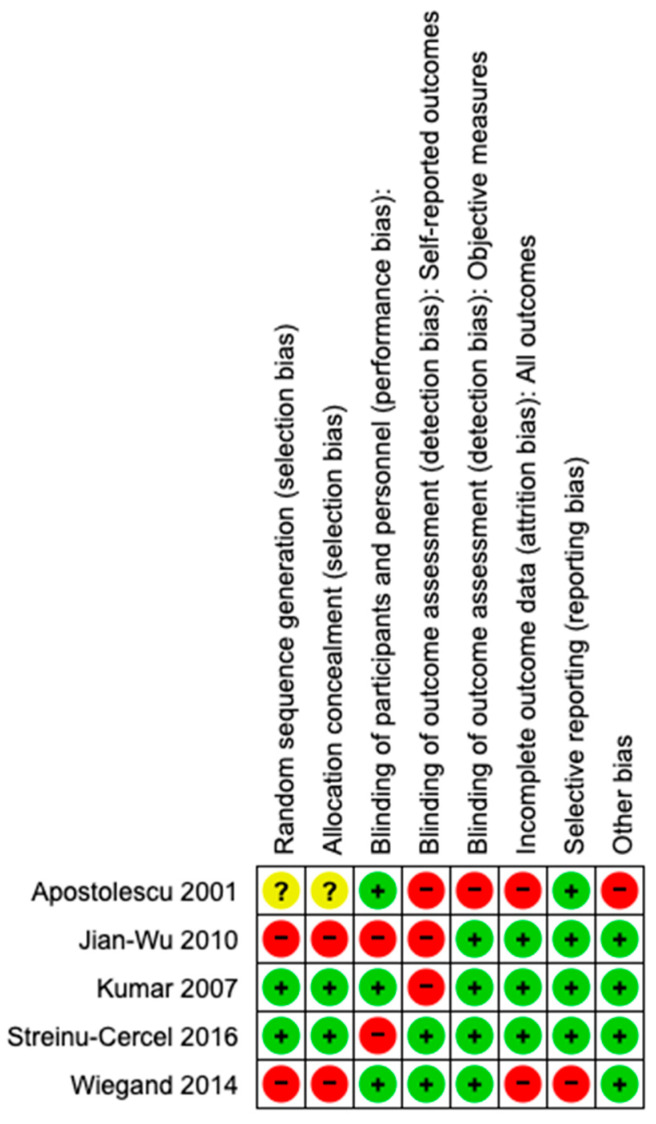
Risk of bias summary based on authors’ judgements about each risk of bias item for each included study [20,21,22,23,24].

Risk of bias: Among five trials, only two reported adequate methods of allocation concealment and random sequence generation. Only three trials were blinded for the participants, and two of them were blinded for the outcome assessment. Nevertheless, the lack of blinding may not have affected the results since the primary outcome (serological cure and death) was objectively measured. One trial [20] was considered at high risk of bias because it did not provide enough information to assess the attrition bias and was classified as having an unclear risk of bias. One trial [23] was classified as having low risk of bias. See Figure 2 and Figure 3. The risk of publication bias was not determined due to the low number of studies.

### 3.5. Effects of Interventions

The outcomes (serological cure and adverse events) were communicated in all reports, excepting two trials [20,22] that did not report side effects. Most trials recruited fewer patients than initially planned due to a drop in the incidence of hepatitis B related to vaccination programs and underdetection of new asymptomatic cases.

**Figure 4:**
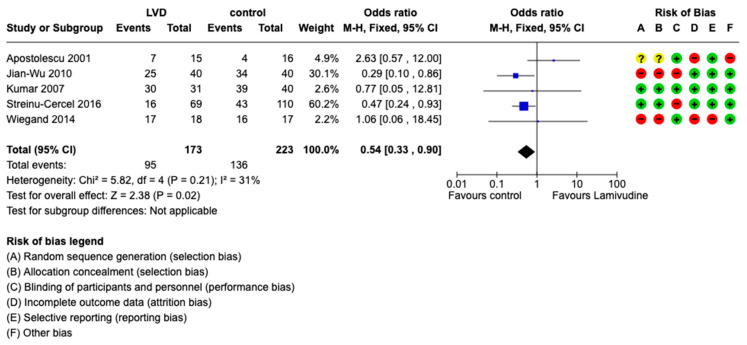
Comparison between lamivudine vs. control for acute hepatitis B virus infection. Outcome: Seroconversion to negative of HBsAg [20,21,22,23,24].

**Figure 5:**
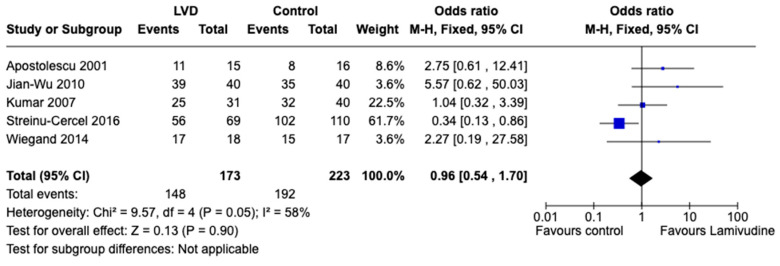
Comparison between lamivudine vs. control for acute hepatitis B virus infection. Outcome: Undetectable HBV DNA [20,21,22,23,24].

All included trials measured the virological response at different follow-up periods (weekly or monthly for 18 months). The virological response was measured as undetectable rate of HBV DNA or reduction in the viral load [24]. Seroconversion of HBsAg to negative favored placebo/standard-of-care compared to lamivudine: OR 0.54, 95% CI 0.33 to 0.9 (*p* = 0.02), I2 = 31%, 396 participants, five trials (Figure 4). Virological cure did not favor any intervention: OR 0.96, 95% CI 0.54 to 1.7 (*p* = 0.90), I2 = 58%, 396 participants, five trials (Figure 5).

**Figure 6:**
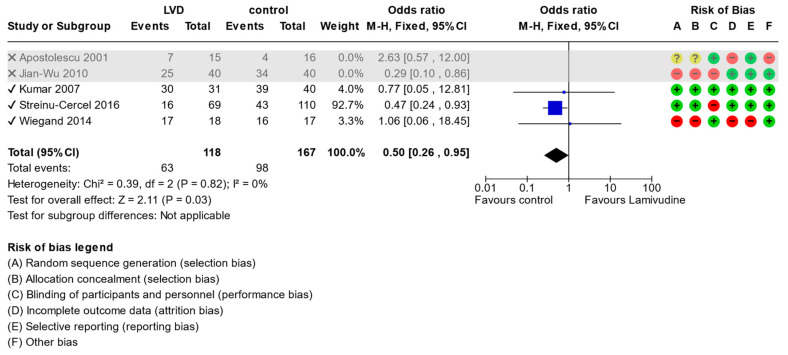
Comparison (excluding studies with high risk of bias) between lamivudine vs. control for acute hepatitis B virus infection. Outcome: Seroconversion to negative of HBsAg [20,21,22,23,24].

**Figure 7:**
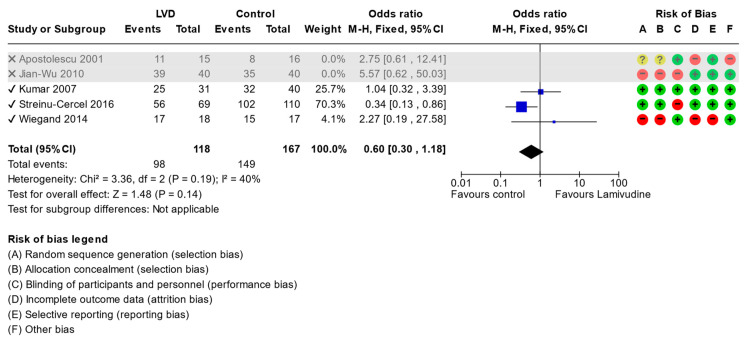
Comparison (excluding studies with high risk of bias) between lamivudine vs. control for acute hepatitis B virus infection. Outcome: Undetectable HBV DNA [20,21,22,23,24].

The subgroup analysis with the exclusion of trials with high risk of bias [20,22] had no impact on the seroconversion of HBsAg when lamivudine was compared with standard-of-care (OR: 0.5, 95% CI 0.26 to 0.95, *p* = 0.03) (Figure 6) or undetectable HBV DNA (OR:0.6, 95% CI 0.30 to 1.18, *p* = 0.14) (Figure 7).

**Figure 8:**
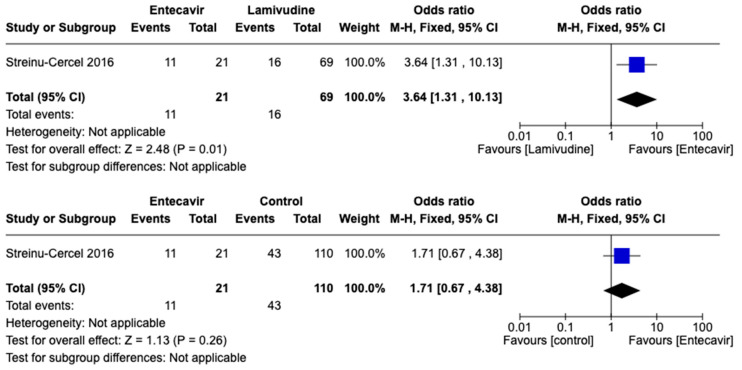
Comparison of entecavir versus lamivudine and entecavir versus standard-of-care for acute hepatitis B virus infection. Outcome: Seroconversion to negative of HBsAg [24].

The only trial that compared entecavir and lamivudine [24] in terms of seroconversion of HBsAg favored entecavir over lamivudine (OR:3.64, 95% CI 1.31–10.13; 90 participants), but entecavir was not favored compared to standard-of-care (OR:1.71, 95% CI 0.67–4.38; 131 participants). See Figure 8.

There was a difference in the reported biochemical recovery (transaminases or total bilirubin). One trial [24] reported normalization of total bilirubin at 24 weeks in nineteen (27.5%) patients treated with lamivudine, four (19%) with entecavir, and twenty-five (22.7%) with standard-of-care. Normalization of ALT at 24 weeks was achieved in 16 (23%) patients treated with lamivudine, 10 (47.6%) with entecavir, and 38 (34.5%) with standard-of-care. Yu et al. reported normalization of total bilirubin at 12 weeks in 37 (92.5%) patients treated with lamivudine and in 30 (75%) with standard-of-care. Other trials [21,23] reported bilirubin and transaminases as changes in levels over time.

### 3.6. Mortality

One trial [20] did not report the mortality data. Two trials [21,23] reported no deaths during the trial or follow-up periods. One trial [22] reported 13 patients that died (3/40 in the lamivudine group and 10/40 in the standard-of-care group) after three months. One trial [24] reported 10 patients who died at one year follow-up (4/69 in the lamivudine group, 1/21 in the entecavir, and 5/110 in standard-of-care group, respectively). The deaths were related to the underlying liver disease.

### 3.7. Adverse Events

Only three trials [21,23,24] reported mild adverse events, and only one trial [23] reported two serious adverse events (depression and gastroenteritis) that were unrelated to the study intervention. One trial [24] reported nausea and vomiting in patients of three groups (lamivudine, entecavir, and standard-of-care). None of the patients stopped therapy or discontinued normal daily activities.

## 4. Discussion

This review aimed to assess the effectiveness of lamivudine and entecavir in the virological cure of adult non-compromised patients with acute severe primary hepatitis B viral infection. Only five studies met the inclusion criteria. There was no additional evidence since the last meta-analysis published in 2017. Thus, there was no further interest in studying NA in acute VHB. This lack of interest is probably related to low prevalence of acute HBV infections, high rate of clinical and virological recovery without antiviral treatment, and widely extended vaccination coverage in the developed countries. In our review, most trials were conducted before 2009, with higher prevalence of HBV infection. Nevertheless, hepatitis B is still an endemic public health issue in the developing countries, with high mortality and morbidity worldwide [32].

These findings are important from the clinical perspective. This review does not allow the formulation of clear treatment recommendations, due to the low quality of evidence, but based on the scarce published data, lamivudine is not effective for the treatment of acute severe HBV infection. To date, the guidelines recommend antiviral treatment for severe acute HBV infection (coagulopathy or protracted course) or acute liver failure [33,34,35,36]. Some of the guidelines point out antiviral treatment preferences such as entecavir, tenofovir disoproxil fumarate (TDF), or tenofovir alafenamide (TAF). However, there is only one trial supporting the use of entecavir for severe acute HBV infection, and no trials have tested TDF or TAF.

This review does not provide information on the ideal doses for different ages. Lamivudine was used in all trials at the same doses, with variations in the time of treatment, but with the same outcome. We cannot answer the question regarding the benefit of nucleosides in selected patients (younger, elderly, immunocompromised, fulminant hepatitis), as most of the trials did not include additional information. Immunocompromised people are the most vulnerable population at risk of developing fatal illnesses. Unfortunately, this review provides little information about the effects of treatment in this population. About the chronicity of HBV, the trials included in this systematic review were not primarily designed to evaluate the effectiveness of nucleosides in preventing the chronic stage, but they determined the seroconversion of HBsAg as a marker of cure and no progression to chronic stage in the study population, which may be considered representative of the general risk population.

Nausea and vomiting were the most frequent mild adverse events reported, and only two serious adverse events were notified. In general, adverse events were poorly assessed. No abnormal laboratory test results were reported in patients who received lamivudine or entecavir. Thus, NA seem to be well tolerated and safe in patients with acute viral hepatitis, although acute liver failure was not established in the included population.

Many trials did not adequately report the trial characteristics which are relevant for evaluating the quality of the evidence. Most trials did not explain whether or how the sample size was predetermined, and many had small sample sizes. Almost none of the trials used an adequate method for allocation concealment or blindness. However, we have considered that lack of blindness constituted a low risk of bias since the measurement of the outcome (seroconversion of HBsAg and undetectable HBV DNA) was performed objectively. In addition, there was insufficient information to assess the attrition bias in the included trials.

Regarding potential biases in the review process, publication bias is a major threat to the validity of systematic reviews. To minimize the risk of publication bias, we conducted a comprehensive search of numerous clinical trial databases. Nonetheless, as in any systematic review, we cannot rule out the influence of publication bias. Unfortunately, given the small number of included trials, we were unable to reliably assess the presence of the publication bias. The low certainty of the evidence obtained in GRADE is another limitation based in the high risk of bias and the heterogeneity of the results across studies.

Finally, we identified a Cochrane systematic review published in 2017 [15] on the treatment of acute HBV infection. This systematic review included the same four studies, excluding the trial of Jian Wu Yu et al. [22] (quasi-randomized trial). We have not identified other randomized trials regarding the treatment of acute HBV infection. Although this was a quasi-randomized trial, the population, inclusion criteria, and outcome variables were similar to those of other trials. Even if selection bias was introduced in our systematic review, the subgroup analysis did not significantly change the outcome, the methods were similar to those of the other trials, and the results were consistent with those of other trials.

The results of our review do not differ from those of Mantzoukis et al. [15] regarding the lack of benefit of seroconversion of HBsAg or obtaining undetectable HBV DNA with lamivudine, compared to standard-of-care in treating patients with severe acute HBV infection.

Lamivudine should not be recommended for the treatment of patients with acute HBV infection. Entecavir has been shown to be superior in only one trial and is preferred to TDF/TAF in the guidelines for the treatment of acute HBV infection. Since some trials [22,24] suggest that treatment with NA compared to standard-of care may decrease mortality in patients with underlying liver disease, further clinical trials are needed to define the role of NA in severe cases.

## 5. Conclusions

The results suggest that there is insufficient evidence that both NA obtain superior results in terms of efficacy (virological cure and HBsAg seroconversion) compared with placebo/standard-of-care in patients with acute viral hepatitis. Subgroup analyses revealed no differences in the lack of efficacy of NA, excluding trials with high risk of bias.

## Figures and Tables

**Table 1:** Characteristics of included trials.

Reference	Settings	Study Period	Study Design	Study Population	Median Age	Female %
Apostolescu 2001 [20]	Romania	2000	RCT double blind: 15 LVD and 16 not	31	No reported	No reported
Kumar 2007 [21]	India	January 2002 to March 2005	RCT double blind: 31 LVD and 40 placebo	71	LVD: 37.2 plac: 36.4	LVD: 35.5 plac: 20
Yu 2010 [22]	China	September 2006 to May 2008	Consecutive hospitalized patients randomly assigned in a 1:1 ratio according to the admission order: 40 LVD and 40 SOC	80	LVD: 45.8 SOC: 44.5	LVD: 25 SOC: 28
Wiegand 2014 [23]	Germany	December 2006 to December 2008	RCT multicentre (24 centres) double blind: 18 LVD and 17 placebo	35	LVD: 39 plac: 42	LVD: 6 plac: 23
Streinu-Cercel 2016 [24]	Romania	May 2005 to May 2009	Open-label RCT: 69 LVD, 21 ECV and 110 SOC	200	LVD: 37.9 ECV: 34.8 SOC: 35.2	LVD: 42 ECV: 49 SOC: 62

RCT: Randomized Clinical Trial. LVD: Lamivudine. ECV: Entecavir. SOC: Standard-of-care.

**Table 2:** Recruitment and following period of included trials.

Reference	Recruitment	Following Period	Treatment	Viral Markers	Mortality
Apostolescu 2001 [20]	Early stages of acute viral hepatitis	3 months	LVD 100 mg daily	HBsAg, HBs Ab, HBcAb-IgM and IgG, HBeAg, and HBV DNA	No reported
Kumar 2007 [21]	Acute viral hepatitis (ALT and TB more than 2.5 times the upper limit, IgM anti HBc positive.	12 months	LVD 100 mg daily for 3 months	HBsAg, HBs Ab, HBcAb-IgM, HBeAg, HBeAb and HBV DNA	No mortality
Yu 2010 [22]	Acute viral hepatitis: TB > 171 umol/L, INR 1.4–1.6, HBsAg, HBV DNA > 1 × 10^4^ copies/mL, ALT more than 5 times the upper limit, HBcAb IgM positive, and HBeAb negative	3 months	LVD 100 mg daily until HBsAg was cleared	HBsAg, HBs Ab, HBcAb, HBeAg, HBeAb and HBV DNA	3 died in the LVD group and 10 died in the SOC group
Wiegand 2014 [23]	Acute viral hepatitis: TB > 85 umol/L, prothrombin time > 50% of normal, HBsAg, HBV DNA > 1 × 10^4^ copies/mL, ALT more than 10 times the upper limit, HBcAb IgM positive	6 months	LVD 100 mg daily until 4th week after loss of HBsAg or for a maximum of 24 weeks	HBsAg, HBs Ab, and HBV DNA	No reported
Streinu-Cercel 2016 [24]	Acute viral hepatitis: TB > 85 umol/L, prothrombin time < 36% or INR > 2, HBsAg, HBV DNA > 1 × 10^4^ copies/mL, ALT more than 5 times the upper limit, HBcAb IgM positive	6 months	LVD 100 mg daily ECV 0.5 mg daily for a maximum of 24 weeks	HBsAg, HBs Ab, HBcAb, HBeAg, HBeAb and HBV DNA	4 died in the LVD group, 1 in the ECV group and 5 in the SOC group

LVD: Lamivudine. ECV: Entecavir. SOC: Standard-of-care. TB: Total Bilirubin. ALT: Alanine aminotransferase. INR: International Normalized Ratio. HBsAg: Hepatitis B surface antigen. HBsAb: Hepatitis B surface antibody. HBcAb: Hepatitis B core antibody. HBeAg: Hepatitis B e antigen. HBeAb: Hepatitis B e antibody.

## Data Availability

The datasets used and/or analyzed during the current study is available from the corresponding author on reasonable request.

## Supplementary Materials

The following supporting information can be downloaded at: https://www.mdpi.com/article/10.3390/v15112241/s1, Table S1: Searching strategy; Table S2: Summary of findings for the main comparison. Lamivudine vs. no intervention for acute hepatitis B virus infection; Table S3: Summary of findings. Entecavir vs. Lamivudine for acute hepatitis B virus infection; Table S4: Summary of findings. Entecavir vs. no intervention for acute hepatitis B virus infection.
