# Supplementary material for: Lamivudine and Entecavir for Acute Hepatitis B: A Systematic Review and Meta-Analysis
Source: Viruses. 2023 Nov 10;15(11):2241. doi: 10.3390/v15112241 (PMC10675181; doi:10.3390/v15112241)
Supplement: Supplementary file 1 [file viruses-15-02241-s001.zip › viruses-2664263-supplementary.pdf]

Supplementary Materials

Table S1. Searching strategy

Pubmed (OVID)	EMBASE
1. exp Hepatitis B	1. hepatitis B
2. acute.tw.	2. acute
3. 1 and 2	3. 1 and 2
4. randomized controlled trial.pt.	4. randomized controlled trial
5. controlled clinical trial.pt.	5. controlled clinical trial
6. lamivudine	6. lamivudine
7. nucleoside	7. nucleoside
8. 4 or 5 or 6 or 7	8. 4 or 5 or 6 or 7
9. 3 and 8	9. 3 and 8

Table S2. **Summary of findings for the main comparison. Lamivudine vs no intervention for acute hepatitis B virus infection**

Lamivudine compared to standard of care for acute hepatitis B infection. **Setting: inpatients**

Certainty assessment							№ of patients		Effect		Certainty
№ of studies	Study design	Risk of bias	Inconsistency	Indirectness	Imprecision	Other considerations	lamivudine	standard of care	Relative (95% CI)	Absolute (95% CI)	
**Undetectable HBV DNA**											
5	randomised trials	serious	serious	not serious	not serious	none	148/173 (85.5%)	192/223 (86.1%)	**OR 0.96** (0.54 to 1.70)	**5 fewer per 1000** (from 91 fewer to 52 more)	⊕⊕◯◯ [1, 2] Low
**Seroconversion HBsAg**											
5	randomised trials	serious	serious	not serious	not serious	none	95/173 (54.9%)	136/223 (61.0%)	**OR 0.54** (0.33 to 0.90)	**152 fewer per 1000** (from 270 fewer to 25 fewer)	⊕⊕◯◯ [1, 2] Low
**Mortality**											
4	randomised trials	serious	serious	not serious	not serious	none	7/158 (4.4%)	15/207 (7.2%)	**OR 0.54** (0.21 to 1.36)	**32 fewer per 1000** (from 56 fewer to 24 more)	⊕⊕◯◯ [1, 2] Low

CI: confidence interval; **OR:** odds ratio; HBV: Hepatitis B Virus; HBsAg: Hepatitis B surface Antigen; SOC: Standard of Care

GRADE Working Group grades of evidence
High quality: Further research is very unlikely to change our confidence in the estimate of effect.
Moderate quality: Further research is likely to have an important impact on our confidence in the estimate of effect and may change the estimate.

Low quality: Further research is very likely to have an important impact on our confidence in the estimate of effect and is likely to change the estimate.
Very low quality: We are very uncertain about the estimate.

[1] The risk of bias in the trials was high (downgraded by 1 level for risk of bias).

[2] Moderate heterogeneity in results across studies (downgrade the quality of evidence for this outcome by 1 level).

Table S3. **Summary of findings. Entecavir vs Lamivudine for acute hepatitis B virus infection**

Entecavir compared to Lamivudine for acute hepatitis B infection **Setting: inpatients**

Certainty assessment							№ of patients		Effect		Certainty	
**№ of studies**	**Study design**	**Risk of bias**	**Inconsistency**	**Indirectness**	**Imprecision**	**Other considerations**	**[Entecavir]**	**[Lamivudine]**	**Relative (95% CI)**	**Absolute (95% CI)**		
**Seroconversion HBsAg**												
1	randomised trials	serious	not serious	not serious	not serious	none	16/69 (23.2%)	11/21 (52.4%)	**OR 3.64** (1.31 to 10.13)	**276 more per 1,000** (from 67 more to 394 more)	⨁⨁⨁◯[1] Moderate	
**Reduction viral load**												
1	randomised trials	serious	not serious	not serious	not serious	none	14/21 (66.7%)	56/69 (81.2%)	**OR 0.46** (0.16 to 1.38)	**147 fewer per 1,000** (from 404 fewer to 44 more)	⨁⨁⨁◯[1] Moderate	

CI: confidence interval; **OR:** odds ratio; HBsAg: Hepatitis B surface Antigen

[1] The risk of bias in the trials was high (downgraded by 1 level for risk of bias).

Table S4. **Summary of findings. Entecavir vs no intervention for acute hepatitis B virus infection**

Entecavir compared to standard of care for acute hepatitis B infection **Setting: inpatients**

Certainty assessment							№ of patients		Effect		Certainty	
**№ of studies**	**Study design**	**Risk of bias**	**Inconsistency**	**Indirectness**	**Imprecision**	**Other considerations**	**[Entecavir]**	**[standard of care]**	**Relative (95% CI)**	**Absolute (95% CI)**		
**Seroconversion HBVsAg**												
1	randomised trials	serious	not serious	not serious	not serious	none	11/21 (52.4%)	43/110 (39.1%)	**OR 1.71** (0.67 to 4.38)	**132 more per 1,000** (from 90 fewer to 347 more)	⨁⨁⨁◯[1] Moderate	
**Reduction viral load**												
1	randomised trials	serious	not serious	not serious	not serious	none	11/21 (52.4%)	102/110 (92.7%)	**OR 0.16** (0.05 to 0.50)	**256 fewer per 1,000** (from 538 fewer to 63 fewer)	⨁⨁⨁◯[1] Moderate	

CI: confidence interval; **OR:** odds ratio; HBsAg: Hepatitis B surface Antigen

[1] The risk of bias in the trials was high (downgraded by 1 level for risk of bias).
